# Development and application of a multidimensional instrument to evaluate competency discrepancies in orthodontic practice

**DOI:** 10.1186/s12903-026-08237-2

**Published:** 2026-04-06

**Authors:** Chiyuen Cheung, Hongfei Lu, Shaoqin Tu, Xinyuan Chen, Yi Feng, YiTing Shao, Yuyao Liu, Zhihui Mai, Hong Ai, Zheng Chen

**Affiliations:** Department of Stomatology, Third Affiliated Hospital of Sun Yat-sen University, 600#, Tianhe Road, Guangzhou, Guangdong P.R. China

**Keywords:** Orthodontists, Multidisciplinary Competency, Scale Development, Cross-sectional study, Clinical Competency Assessment

## Abstract

**Background:**

Contemporary orthodontic practice faces challenges related to treatment complications, often linked to deficiencies in multidisciplinary clinical competency. A clear understanding of the coordination across the stomatognathic system is essential, yet no standardized instrument exists to assess core skills. This study aimed to develop and validate a comprehensive assessment tool and evaluate current competency levels and associated factors among Chinese orthodontists across dental, periodontal, temporomandibular joint (TMJ), and myofunctional domains.

**Methods:**

A cross-sectional survey was conducted across multiple regions of China from January to June 2025 among 434 practicing orthodontists. A three-component assessment instrument was developed through systematic expert review: Subjective Competency Scale (SCS), Clinical Practice Behavior Scale (CPBS), and Objective Knowledge Test for Orthodontists (OKTO). Psychometric properties were evaluated through exploratory factor analysis, reliability testing, and validity assessment. Competency variations across four domains were analyzed using Friedman tests, and demographic predictors were identified through stepwise multiple regression.

**Results:**

The instrument demonstrated satisfactory reliability (SCS: Cronbach’s α = 0.897) and validity. Significant competency disparities existed across domains (all *p* < 0.001). Orthodontists showed highest competencies in dental areas (SCS: 4.45 ± 0.77; practice compliance: 78.00%) but substantial deficits in TMJ (SCS: 3.33 ± 1.17; practice compliance: 56.45%) and myofunctional domains. Objective knowledge patterns revealed adequate TMJ knowledge (82.58%) but poor myofunctional understanding (68.26%). Educational attainment emerged as the strongest predictor (β = 0.262, *p* < 0.001), followed by age (β = 0.201) and continuing education participation (β = 0.127).

**Conclusions:**

This study represents an initial attempt to develop and validate a multidimensional competency assessment framework for orthodontists, with the aim of offering a structured approach to evaluating interdisciplinary clinical competencies. TMJ and myofunctional domain-specific deficiencies, which were found to be associated with educational attainment, may serve as potential risk factors affecting treatment stability and complication prevention within the Chinese clinical context. This instrument, with preliminary validation evidence based on a Chinese sample, offers a tentative framework for systematic competency evaluation that requires further validation across diverse contexts.

**Supplementary Information:**

The online version contains supplementary material available at 10.1186/s12903-026-08237-2.

## Introduction

Malocclusion, defined as any deviation from ideal occlusion that compromise function, esthetics, or periodontal health, represents one of the most prevalent global oral health conditions [1], affecting 56–87% of populations worldwide and significantly impacting quality of life [2, 3]. Over nearly a century of orthodontic development, the field has witnessed remarkable technological advances—from Angle’s Edgewise appliance to Andrews’ Straight-wire technique, and more recently to lingual orthodontics and clear aligner therapy [4]. These innovations have progressively expanded treatment capabilities, enabling orthodontists to successfully manage increasingly complex malocclusions that were once considered untreatable.

Despite these substantial technological achievements, contemporary orthodontic practice continues to face serious clinical challenges. Treatment-related complications remain prevalent, including orthodontically-induced white spot lesions [5], gingival recession and periodontal attachment loss [6], temporomandibular disorders [7] and myofunctional/orofacial dysfunction including persistent oral habits and breathing pattern disorders [8]. These complications significantly compromise treatment outcomes and can substantially diminish long-term stability.

While patient-related factors—such as inadequate oral hygiene compliance and genetic predisposition—undoubtedly contribute to these adverse events, an equally critical question emerges: do these complications also reflect deficiencies in clinician competencies? specifically, do orthodontists possess comprehensive knowledge systems necessary for appropriate case selection during initial diagnosis, vigilant monitoring with continuous patient education throughout treatment, and adequate provision of suitable long-term maintenance protocols? Currently, no standardized instrument exists to systematically assess orthodontists’ multidisciplinary competencies, identify specific knowledge gaps, and guide targeted professional development to address this pressing clinical challenge of treatment-related complications.

Addressing these quality concerns requires a clear understanding of what constitutes competent orthodontic practice in the modern era. With the rising need for orthodontic care, modern orthodontic practice has evolved beyond simple tooth alignment to focus more critically on the coordinated balance of all components within the Stomatognathic system [9], to achieve the ideal goals of aesthetics, function, and stability [1]. Dental and periodontal health are critical for achieving optimal aesthetic results and preventing treatment-related complications [10, 11]. Meanwhile, temporomandibular joint and myofunctional health are essential for maintaining proper functional balance and long-term treatment stability, as dysfunction in these areas can disrupt the entire biomechanical equilibrium and significantly increase relapse risk [12, 13]. From a biomechanical perspective, the head-neck region, respiratory system, and masticatory system constitute an interdependent functional unit where dysfunction in any component can trigger cascading effects throughout the entire system [14].Therefore, we considered that a comprehensive assessment of orthodontist competencies across these four domains—dental, periodontal, temporomandibular, and myofunctional—is essential for evaluating their ability to manage this complex biomechanical system and deliver high-quality, sustainable orthodontic care.

In China, the challenge of ensuring orthodontic quality is particularly acute. Driven by high malocclusion prevalence and increasing aesthetic awareness, China’s orthodontic market has experienced rapid growth. However, a significant mismatch exists between the quantity and quality of Chinese orthodontists relative to market demand. As noted by Zhou et al., China’s current health inequity is due less to doctor shortage but more to abundant yet poorly trained health professionals [15], the average orthodontic patient satisfaction across China remains moderate, with satisfaction levels showing significant regional variations [16].These characteristics make China an ideal context for developing and validating a comprehensive competency assessment tool: the combination of high treatment demand, large number of clinical practitioners, and treatment quality concerns. Based on this rationale, we developed and validated a comprehensive assessment instrument specifically designed to evaluate multidisciplinary treatment competencies among Chinese orthodontists. The study objectives were to: (1) develop a comprehensive multidisciplinary treatment (MDT) competencies assessment tool; (2) evaluate its reliability and validity; (3) assess current multidisciplinary competency levels among Chinese orthodontists; and (4) identify factors associated with competency variations to inform educational and professional development initiatives.

Using this preliminarily validated instrument, we conducted a nationwide survey and identified significant competency gaps, particularly in the temporomandibular joint and myofunctional domains, and identified educational background as the primary factor influencing these variations. This instrument, preliminarily validated within the Chinese context, may serve as an initial framework adaptable across different healthcare systems.

## Methods

### Study design and participants

This cross-sectional survey study was conducted from January to June 2025 to assess multidisciplinary competencies among Chinese orthodontists. The study was designed according to the STROBE guidelines for cross-sectional studies.

Practicing orthodontists were recruited nationwide through a two-stage approach. First, we sent email and/or text messages with a survey link to respondents who had attended orthodontic continuing education programs at various major tertiary hospitals in China, as well as orthodontic clinical fellows and residents in relevant standardized training programs (all programs recruit participants nationally from across China). Second, we invited members of several national orthodontic professional WeChat groups to complete the questionnaire. All questionnaires were administered online via the Wenjuanxing platform (www.wjx.cn). To improve response rates, participants were encouraged to share the survey link within their professional WeChat groups. No additional incentives were provided. A total of 1,479 online questionnaire invitations were distributed via email and WeChat. All participants were informed of voluntary participation and data confidentiality.

Participants were required to be practicing orthodontists with a minimum of six months of clinical experience. No restrictions were placed on practice setting or geographic location. Participants were excluded if they: (1) completed the survey in less than 180 s; (2) failed either of the two attention check questions verifying basic orthodontic knowledge; or (3) submitted duplicate responses.

### Instruments

The instrument was developed using a systematic 2-round review procedure. In the first round, current orthodontic practice guidelines and scientific literature were reviewed (Supplementary File 2), and discussions among 5 orthodontic experts (H.A. and 4 other experienced orthodontists) were conducted to develop the initial instrument framework. The instrument was designed to assess competencies across four domains—dental, periodontal, TMJ, and myofunctional—with items specifically developed to evaluate skills across the entire treatment continuum: pre-treatment risk assessment, intra-treatment monitoring, and post-treatment follow-up care.

In the second round, a panel of multidisciplinary experts (3 orthodontists, 2 periodontists, 2 oral and maxillofacial surgeons, and 1 temporomandibular joint specialist) reviewed the instrument for clarity, face validity, and content validity. Based on their feedback, the instrument was refined to obtain the final version. The final instrument consisted of three integrated components including Subjective Competency Scale (SCS), Clinical Practice Behavior Scale (CPBS) and Objective Knowledge Test for Orthodontists (OKTO).

Two fundamental orthodontic knowledge questions were included as attention checks and to ensure respondents possessed basic orthodontic expertise. Questionnaires with incorrect answers to these questions were excluded from the analysis.

Detailed Instrument development procedures are provided in Supplementary File 1.

### Statistical analysis

The analysis followed a systematic framework: (1) descriptive statistics for participant characteristics and scale scores; (2) psychometric evaluation including exploratory factor analysis and confirmatory factor analysis for SCS, item analysis for OKTO, and reliability assessment using Cronbach’s alpha for SCS and Kuder-Richardson 20 (KR-20) for OKTO; (3) validity testing through correlation analysis and known-groups validation using CPBS tertile groups; (4) competency assessment comparing four dimensions using Friedman tests and demographic group differences using appropriate non-parametric tests; and (5) multiple regression analysis to identify predictors of MDT competency. Age and years of orthodontic practice were treated as ordinal categorical variables in all analyses, consistent with their collection format in the survey. All survey items were set as mandatory in the online platform, ensuring complete responses from all included participants. No missing data were present in the final analytical sample.

SCS and CPBS were scored as mean values (range 1–5), while OKTO was scored as total correct responses (range 0–19). A composite Multidisciplinary Treatment (MDT) score was calculated as: MDT = 0.3×SCS + 0.4×CPBS + 0.3×OKTO. The weighting scheme was based on Miller’s pyramid of clinical competence(Miller, 1990), prioritizing CPBS while maintaining balanced representation of perceived competency and objective knowledge. Descriptive statistics included means ± standard deviations (SD) and interquartile ranges (IQR) for continuous variables, and frequencies for categorical variables. Test-retest reliability was assessed in a subset of 35 participants who completed the instrument twice with an interval of 2 to 4 weeks. Intraclass correlation coefficients (ICC) were calculated using a two-way mixed-effects model with absolute agreement, reporting single measures ICC with 95% confidence intervals.

Spearman correlations examined relationships between components and demographic variables. Participants were stratified by CPBS scores (tertiles) for known-groups validity testing using Kruskal-Wallis tests with Dunn’s post-hoc comparisons. Friedman tests with Nemenyi post-hoc comparisons compared the four competency dimensions (dental, periodontal, temporomandibular joint, and myofunctional) within scales. Chi-square tests examined associations between demographic characteristics and MDT competency groups. Stepwise multiple regression identified predictors of MDT competency.

## Result

**Fig. 1:**
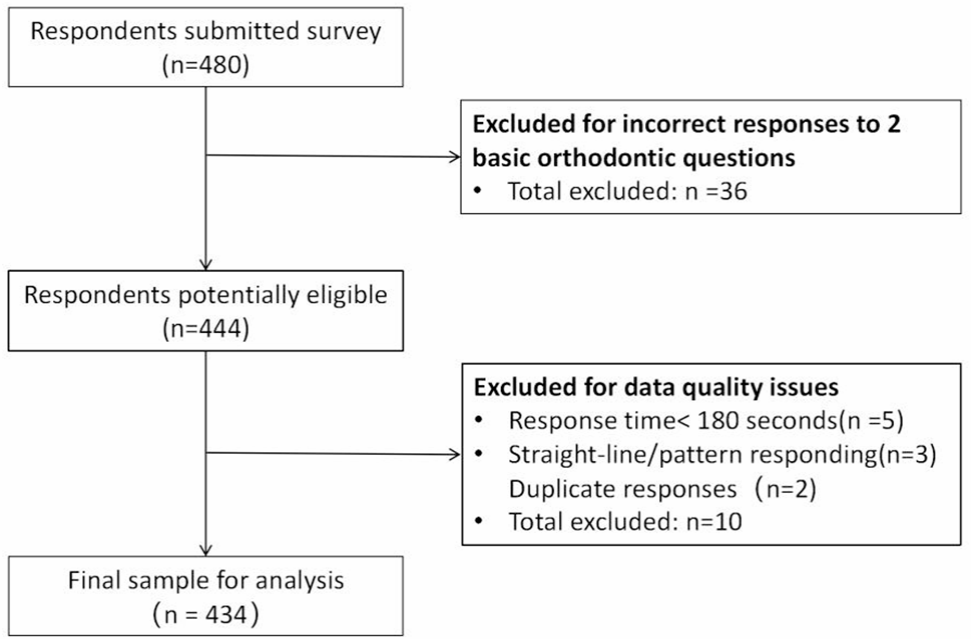
Flowchart demonstrating the selection process of study participant

A total of 480 respondents submitted surveys, and 434 met the selection criteria and were included in the analyses(Fig. 1), with a predominance of female practitioners. 5. Of the 46 excluded respondents,5 were removed for completing the survey in less than 180 s, 36 failed the first basic orthodontic knowledge check question(D1), 3 failed the second check question(D2), and 2 submitted duplicate responses.The sample size of 434 was considered adequate for the planned analyses: exploratory factor analysis generally requires a minimum of 5–10 participants per item [17], and with 12 SCS items, our sample exceeds this threshold; multiple regression analysis with three predictors requires approximately 50–100 participants at minimum, which our sample substantially surpasses [18]. The majority of participants were aged 31–40 years and held bachelor’s degrees or master’s degrees. Geographically, over half of the respondents practiced in Eastern China. Regarding professional background, 40.09% specialized in orthodontics while 53.92% were general dental practitioners. Practice settings varied widely, with private dental clinics and tertiary A-level general hospitals being the most frequent workplaces. The majority attended 1–3 continuing education courses in the previous two years. Traditional fixed appliance treatment was the most common professional strength, followed by early childhood orthodontics and clear aligner treatment(Table 1 ).

**Table 1:** Demographic characteristics of participants

Characteristic	Respondents in Current Study (*N* = 434) (no. [%])
Sex	
Male	167 (38.48%)
Female	267 (61.52%)
Age Group	
18–25	16 (3.69%)
26–30	58 (13.36%)
31–40	205 (47.24%)
41–50	107 (24.65%)
51–60	41 (9.45%)
Over 60	7 (1.61%)
Practice Location	
Eastern China	255 (58.76%)
Central China	63 (14.52%)
Western China	106 (24.42%)
Northeast China	10 (2.30%)
Educational level	
Associate Degree	55 (12.67%)
Bachelor’s Degree	193 (44.47%)
Master’s Degree	141 (32.49%)
Doctoral Degree	45 (10.37%)
Professional Specialty	
Dental Medicine (General)	174 (53.92%)
Orthodontics	234 (40.09%)
Endodontics	8 (1.84%)
Implant Dentistry	2 (0.46%)
Periodontics	3 (0.69%)
Oral & Maxillofacial Surgery	5 (1.15%)
Pediatric Dentistry	7 (1.61%)
Preventive Dentistry	1 (0.23%)
Job Title	
Resident Physician	105 (24.19%)
Attending Physician	194 (44.7%)
Associate Chief Physician	97 (22.35%)
Chief Physician	38 (8.76%)
Years of practice in orthodontics	
< 1 Year	59 (13.59%)
1–3 Years	103 (23.73%)
4–6 Years	59 (13.59%)
7–10 Years	83 (19.12%)
11–20 Years	77 (17.74%)
> 20 Years	53 (12.21%)
Workplace	
Private Dental Clinic	136 (31.34%)
Chain Dental Institution	81 (18.66%)
Community Hospital	9 (2.07%)
Secondary A-level General Hospital (Dental Department)	24 (5.53%)
Secondary A-level Dental Hospital	17 (3.92%)
Tertiary A-level General Hospital (Dental Department)	96 (22.12%)
Tertiary A-level Dental Hospital	71 (16.36%)
New orthodontic cases per year	
< 20 Cases	141 (32.49%)
21–50 Cases	99 (22.81%)
51–100 Cases	90 (20.74%)
100–200 Cases	64 (14.75%)
> 200 Cases	40 (9.22%)
Orthodontic-Related Continuing Education Courses in Recent 2 Years	
0 Times	22 (5.07%)
1–3 Times	201 (46.31%)
4–6 Times	101 (23.27%)
> 6 Times	110 (25.35%)
Professional Strengths (Multiple Choice)	
Traditional Fixed Appliance Treatment	392 (90.32%)
Clear Aligner Treatment	231 (53.23%)
Early Childhood Orthodontics	286 (65.9%)
Adult Orthodontics	232 (53.46%)
Orthognathic-Orthodontic Combined Treatment	68 (15.67%)
Orthodontic-Periodontal Combined Treatment	85 (19.59%)
Orthodontic-Restorative Combined Treatment	97 (22.35%)
Orthodontic-Sleep Apnea-ENT Combined Treatment	42 (9.68%)
Others	18 (4.15%)

### Reliability and validity analysis of the SCS and the OKTO

Exploratory factor analysis of the SCS extracted four factors corresponding to the four theoretical competency dimensions: dental (items A1-A3), periodontal (items A4-A6), temporomandibular joint (items A7-A9), and myofunctional (items A10-A12) competencies(Supplementary File 4 Table 1). The factor structure supported the theoretical framework, with items loading primarily on their expected dimensions. The Kaiser-Meyer-Olkin measure (0.873) and Bartlett’s test (χ² = 3023.250, *p* < 0.001) confirmed sampling adequacy. The overall SCS demonstrated internal consistency of α = 0.897, with dimension-specific reliabilities of α = 0.662–0.903, indicating good internal consistency for the overall scale and its dimensions.CFA confirmed the four-factor structure of the SCS, with acceptable model fit (CFI = 0.933, RMSEA = 0.098, SRMR = 0.048; Supplementary File 4 Table 2).

Item analysis of the OKTO across 19 questions showed difficulty indices ranging from 0.26 to 0.97(Supplementary File 4 Table 3). This indicates the inclusion of both easy and challenging items, making it appropriate for a comprehensive competency assessment. The overall test’s Kuder-Richardson 20 (KR-20) coefficient was 0.46. This value reflects the test’s intentional heterogeneity, as the questions were designed to assess diverse knowledge domains across four different dimensions for evaluating multidisciplinary competency. Items with moderate difficulty and higher discrimination indices (e.g., C11, C13) showed stronger correlations with overall test performance.

### Inter-instrument validation: SCS, CPBS, and OKTO relationships

Correlation analysis revealed a moderate positive correlation between the SCS and CPBS (*r*_*s*_ = 0.576, *p* < 0.01), indicating a strong association between higher self-perceived competency and more frequent self-reported clinical practices(Table 2). In contrast, the OKTO showed a minimal, non-significant correlation with the SCS (*r*_*s*_ = 0.067, *p* > 0.05). Notably, there is a statistically significant negative correlation between the OKTO and CPBS (*r*_*s*_ = − 0.105, *p* < 0.05), implying that higher objective knowledge scores may correlate with less frequent self-reported clinical practices.

**Table 2:** Spearman correlations among competency scales and demographic variables

Variables	SCS Total Score (rₛ)	CPBS Total Score (rₛ)	OKTO Total Score (rₛ)
SCS Total Score	-		
CPBS Total Score	0.576**	-	
OKTO Total Score	0.067	-0.105*	-
Age	0.227**	0.172**	-0.062
Education	0.346**	0.219**	0.253**
Title	0.278**	0.215**	0.023
Experience	0.313**	0.207**	0.135*
Patient Volume	0.274**	0.190**	0.247**
Orthodontic CE courses attended	0.104*	0.173**	0.076

*SCS *Subjective Competency Scale, *CPBS *Clinical Practice Behavior Scale, *OKTO *Objective Knowledge Test for Orthodontists, *CE *Continuing Education. **p < 0.01, *p < 0.05

Known-groups validity was examined by dividing participants into tertiles based on their CPBS scores using 33rd and 67th percentiles(Supplementary File 4 Table 4). As expected, the high-practice group demonstrated significantly higher SCS scores than both the middle and low-practice groups (all *p* < 0.001). However, OKTO scores showed only modest differences across CPBS groups (*p* = 0.017), with pairwise comparisons revealing significant difference only between the middle and high groups (*p* = 0.013), suggesting that objective knowledge may be less directly related to clinical practice behaviors.

### Multidisciplinary competency comparison

Participants demonstrated varying levels of multidisciplinary competency across the four domains. For SCS, dental competencies scored highest, followed by periodontal, myofunctional, and temporomandibular joint competencies, indicating significantly lower confidence in TMJ management(Supplementary File 3 Fig. 1; Supplementary File 4 Table 5). CPBS showed similar patterns(Supplementary File 3 Fig. 2; Supplementary File 4 Table 6). OKTO revealed good performance in dental (82.63%) and TMJ domains (82.58%), moderate performance in periodontal knowledge (73.56%), but weaker myofunctional knowledge (68.26%) (Supplementary File 4 Table 7). Friedman tests showed significant overall differences among the four domains across SCS, CPBS, and OKTO (all *p* < 0.001) (Table 3; Supplementary File 3 Fig. 3). Post-hoc comparisons using Nemenyi tests confirmed the descriptive pattern, with TMJ and myofunctional competencies showing no significant differences between each other (*p* = 0.9) but both scoring significantly lower than dental and periodontal domains. These findings highlight TMJ and myofunctional competencies as priority areas for continuing education and professional development among Chinese orthodontists in this sample.

**Table 3:** Comparison of competency scores across four multidisciplinary domains

Competency Scale	Domain	Median (IQR)	Friedman Test (χ², *p*)
SCS			416.827, < 0.001**
	Dental	4.342 (0.719)	
	Periodontal	4.333 (1.000)	
	TMJ	3.500 (1.333)	
	Myofunctional	3.833 (1.224)	
CPBS			248.186, < 0.001**
	Dental	4.250 (0.500)	
	Periodontal	3.950 (0.800)	
	TMJ	3.763 (1.022)	
	Myofunctional	3.730 (1.154)	
OKTO			535.416, < 0.001**
	Dental	4.000 (1.000)	
	Periodontal	4.000 (2.000)	
	TMJ	4.000 (1.000)	
	Myofunctional	3.000 (1.000)	

*SCS *Subjective Competency Scale, *CPBS *Clinical Practice Behavior Scale, *OKTO *Objective Knowledge Test for Orthodontists, *TMJ *Temporomandibular Joint. OKTO scores represent correct answers per domain (range 0–5 for each domain). Post-hoc Nemenyi tests were performed to examine pairwise differences. For SCS and CPBS, Dental and Periodontal competencies scored significantly higher than Myofunctional and TMJ competencies (*p* < 0.05). For OKTO, Myofunctional knowledge scored significantly lower than all other domains (all *p* < 0.01), while Dental showed significant differences from Periodontal (*p* = 0.009) and TMJ showed significant differences from Periodontal (*p* = 0.002)

### Factors associated with multidisciplinary competency

Participants were stratified into low, medium, and high MDT competency groups based on the 33rd and 67th percentiles of composite MDT scores (calculated as 0.3×SCS + 0.4×CPBS + 0.3×OKTO), resulting in 167, 136, and 131 participants respectively.

Significant associations were found between demographic characteristics and MDT competency levels (Supplementary File 4 Table 7.). Higher competency correlated with older age, advanced education (e.g., 50.38% of high-MDT group hold postgraduate degrees), senior titles (45.04% deputy/chief physicians), longer years of practice (44.27% >10 years), employment in tertiary hospitals (43.51%), higher patient volumes (33.59% >100 cases annually), and frequent continuing education (58.78% >3 courses in two years).

Stepwise multiple regression analysis identified three independent predictors of MDT competency (R² = 0.14, F(3,430) = 23.356, *p* < 0.001). Educational attainment emerged as the strongest predictor (β = 0.262, *p* < 0.001), followed by age (β = 0.201, *p* < 0.001) and frequency of continuing education participation (β = 0.127, *p* = 0.005). These three factors collectively explained 14% of the variance in MDT competency scores (adjusted R ²= 0.134). Multicollinearity diagnostics indicated acceptable tolerance values (> 0.98) and VIF values (< 1.02), confirming the independence of predictors. The Durbin-Watson statistic (2.044) suggested no significant autocorrelation in residuals. Correlation analysis revealed that educational attainment showed the strongest associations with SCS (*r* = 0.346) and OKTO (*r* = 0.253), while CPBS showed more modest correlations with demographic factors (*r* = 0.172–0.219). An interesting exception was the weak negative correlation observed between age and OKTO (*r* = − 0.062), suggesting that older practitioners’ knowledge may not necessarily be higher (Table 4).

**Table 4:** Stepwise multiple regression analysis of factors associated with MDT competency

Variable	B□	Std. Error	Beta□	t	*p*	VIF□	Tolerance□
Constant	-0.565	0.188	-	-3.008	0.003**	-	-
Age	0.168	0.038	0.201	4.465	0.000**	1.017	0.983
Educational level	0.258	0.044	0.262	5.832	0.000**	1.01	0.99
Orthodontic CE courses (past 2 years)	0.115	0.041	0.127	2.827	0.005**	1.007	0.993

*p < 0.05; **p < 0.01. R² = 0.140; Adjusted R² = 0.134; F(3,430) = 23.356, *p* < 0.001; Durbin-Watson = 2.044. Dependent variable = Weighted MDT competency score. B, unstandardized coefficient; Beta, standardized coefficient; CE, Continuing Education; VIF, Variance Inflation Factor. All tolerance and VIF values indicate no multicollinearity concerns

## Discussion

Malocclusion is a prevalent oral health problem worldwide that significantly impacts patients’ function of stomatognathic system, aesthetics, and quality of life [19, 20]. This study addresses the current status of multidisciplinary treatment competency among Chinese orthodontists. We developed and validated a comprehensive assessment tool covering three parts: subjective competency scale (SCS), clinical practice behavior scale (CPBS), and objective knowledge test (OKTO). The research findings reveal that Chinese orthodontists in this sample demonstrate greater proficiency in dental and periodontal fields, but show relatively weaker performance in temporomandibular joint and myofunctional areas. Additionally, we discovered that MDT capabilities are closely correlated with educational background and continuing education participation.

The assessment tool developed in this study demonstrated acceptable reliability and validity in this initial evaluation. Exploratory factor analysis of the SCS supports a four-dimensional structure (dental, periodontal, TMJ, and myofunctional), with an overall Cronbach’s α of 0.897, indicating high internal consistency. However, the OKTO showed a KR-20 coefficient of 0.46, which reflects the intentional heterogeneity of the test design rather than poor item quality. KR-20 assumes unidimensionality and is most appropriate for tests measuring a single construct; however, the OKTO was explicitly designed to assess knowledge across four distinct clinical domains, and low inter-item correlations across domains are therefore expected. This interpretation is supported by item-level analysis: deletion of any single item produced minimal change in KR-20 (range: 0.39–0.47), confirming that the low coefficient reflects structural heterogeneity rather than problematic items. Furthermore, key discriminating items demonstrated acceptable psychometric properties, with discrimination indices of 0.45–0.46 and point-biserial correlations of 0.47–0.51 for the strongest items (C11, C13). Several items also exhibited ceiling effects (difficulty index > 0.95), which mechanically suppressed the overall KR-20 while retaining descriptive value in characterizing knowledge distribution patterns.Additionally, the significant positive correlation between SCS and CPBS (rs = 0.576) indicates that orthodontists’ self-assessment of capabilities aligns well with their clinical practice behaviors. Interestingly, OKTO showed a weak negative correlation with clinical behavior (rs = − 0.105), suggesting a complex relationship between theoretical knowledge and practical application.

In terms of SCS, orthodontists demonstrated higher confidence in dental (4.45 ± 0.77) and periodontal (4.33 ± 0.81) fields, with scores significantly higher than those in temporomandibular joint (3.33 ± 1.17) and myofunctional (3.79 ± 1.03) areas (*p* < 0.001). This difference in self-perception was further confirmed in CPBS: dental-related procedures showed the highest compliance rate (78.00%), while TMJ-related procedures had the lowest compliance rate (56.45%). Notably, the OKTO results presented a different capability distribution pattern: orthodontists demonstrated better knowledge mastery in dental (82.63%) and temporomandibular joint (82.58%) fields, but showed relatively weaker performance in myofunctional (68.26%) and periodontal (73.56%) areas. This may reflect the increased emphasis on TMJ knowledge education in recent years, as TMD may emerge or be exacerbated during orthodontic procedures, and the TMJ position is considered crucial in establishing ideal occlusal relationships [21–23]. The peculiar phenomenon of “high confidence - high practice - low knowledge” in the periodontal field warrants attention, as it may indicate that some orthodontists’ emphasis on periodontal health management exceeds their actual knowledge base. Besides, only 39.63% of orthodontists routinely perform periodontal probing depth and bleeding point examinations before treatment (B3), though periodontal probing results may provide important insights into the optimal timing for orthodontic treatment in patients with periodontal conditions [24, 25]. Correspondingly, only 68.2% of participants correctly answered questions related to periodontal probing techniques (C6), indicating a concurrent gap in both theoretical understanding and clinical application. Similarly, high-frequency practice rates for intra -treatment monitoring to prevent complications (B7-B10) were all below 50%, suggesting critical preventive practices are often neglected.

Based on correlation analysis and multiple regression models, we found that educational level demonstrated significant positive correlations with SCS, CPBS, and OKTO, making the largest contribution in the regression model. Notably, while the majority of our participants held Associate Degrees (12.67%) and Bachelor’s Degrees (44.47%), these groups demonstrated disproportionately lower MDT competency levels, highlighting the concerning gap between basic education and clinical performance. This indicates that systematic professional education (such as master’s/doctoral training) is associated with higher knowledge levels, greater clinical confidence, and more standardized practice behaviors.Age showed positive correlations with SCS and CPBS but a weak negative correlation with OKTO (rₛ=-0.062). Regression analysis confirmed age as an independent predictor of MDT competency. While age emerged as an independent predictor, it should be noted that age and years of practice represent distinct variables. The association between age and competency may reflect multiple factors beyond clinical experience accumulation, and the independent contribution of practice duration warrants further examination using years of practice as a more direct measure.

Continuing education frequency showed a significant correlation with CPBS but contributed modestly to the regression model (β = 0.127). This suggests that while participation in continuing education is beneficial, the relationship between frequency and clinical competency may be influenced by other factors such as training quality, content relevance, or individual learning preferences. Future research should investigate whether interactive, practice-based continuing education formats are more effective than traditional approaches in enhancing multidisciplinary competencies, as suggested by previous studies [26]. Professional title and annual patient volume were positively correlated with SCS/CPBS, suggesting an association between clinical practice opportunities and competency development.

Currently, many Chinese dental students tend to pursue higher degrees(Yan et al. 2020), driven by the fact that higher qualifications are crucial for career progression and employment opportunities in urban hospitals (Wang X. 2010). This trend may contribute to gradually improving competency levels among orthodontists in Chinese hospitals, though the relationship between degree attainment and clinical quality requires further longitudinal verification. However, increasing reports indicate that China’s healthcare environment is deteriorating, with being a doctor or dentist becoming a dangerous profession, partly because medical quality fails to meet patients’ expectations [27]. To enhance treatment quality, it is crucial to develop comprehensive diagnostic perspectives and multidisciplinary treatment capabilities in the current education system.

Several limitations should be acknowledged. First, participants were recruited through non-probability sampling via email invitations and professional networks, which may limit the generalizability of findings to the broader population of Chinese orthodontists. The sample shows geographic skewing toward Eastern China and overrepresentation of practitioners with continuing education exposure, suggesting that true competency gaps may be larger than those observed in this study. Future studies employing probability-based sampling methods would strengthen the representativeness of findings. The low KR-20 of the OKTO (0.46) represents a limitation of the objective knowledge component. While this is attributable to the test’s multidimensional design, it introduces greater measurement error in the knowledge dimension, which may modestly reduce the precision of the composite MDT score. Given that OKTO contributes 30% to the MDT composite, the overall impact is limited, but future refinement should consider developing domain-specific knowledge subscales with improved internal consistency. The regression model explained only 14% of the variance in MDT competency scores (R² = 0.14), suggesting that other important predictors not captured in this study, such as training quality, mentorship experience, or institutional culture, may also contribute substantially to competency development. Future studies should explore a broader range of potential predictors.

This study provides preliminary directions for improving the professional development of orthodontists, based on findings from a Chinese sample.We recommend integrating multidisciplinary content, particularly temporomandibular joint assessment protocols, myofunctional therapy techniques, and periodontal-orthodontic combined treatment approaches, into residency training programs to reduce competency gaps between different training pathways. Additionally, enhanced continuing education should prioritize high-quality learning activities, particularly targeting private practice clinicians and primary care practitioners who may have limited access to advanced training opportunities. By addressing these educational gaps and standardizing competencies across different training routes, we hope to improve diagnostic quality, enhance multidisciplinary treatment capabilities, and ultimately improve doctor-patient relationships through delivering high-quality medical care in China. Having undergone initial validation among Chinese orthodontists, we hope this tool may serve as a preliminary framework to support standardized competency assessment in orthodontic training programs, pending further validation across diverse populations and healthcare systems.

## Supplementary Information

Supplementary Material 1.

Supplementary Material 2.

Supplementary Material 3.

Supplementary Material 4.

## Data Availability

The data that support the findings of this study cannot be shared publicly due to the privacy of individuals that participated in the study and ethical restrictions. The data will be shared on reasonable request to the corresponding author, subject to ethical approval and privacy agreements.

## Abbreviations

SCS: Subjective Competency Scale
CPBS: Clinical Practice Behavior Scale
OKTO: Objective Knowledge Test for Orthodontists
MDT: Multidisciplinary Treatment
TMJ: Temporomandibular Joint
TMD: Temporomandibular Disorders
EFA: Exploratory Factor Analysis
CFA: Confirmatory Factor Analysis
KR-20: Kuder-Richardson Formula 20
ICC: Intraclass Correlation Coefficient
IQR: Interquartile Range
SD: Standard Deviation
VIF: Variance Inflation Factor
CFI: Comparative Fit Index
RMSEA: Root Mean Square Error of Approximation
SRMR: Standardized Root Mean Square Residual
CE: Continuing Education
