# Supplementary material for: Development and application of a multidimensional instrument to evaluate competency discrepancies in orthodontic practice
Source: BMC Oral Health. 2026 Apr 6;26:775. doi: 10.1186/s12903-026-08237-2 (PMC13134201; doi:10.1186/s12903-026-08237-2)
Supplement: Supplementary file 1 — Supplementary Material 1. [file 12903_2026_8237_MOESM1_ESM.docx]

**Supplementary File 1. Development of Instruments**

The instruments used in this study included four main components: Subjective Competency Scale (SCS), Clinical Practice Behavior Scale (CPBS), Objective Knowledge Test for Orthodontists (OKTO), and questions related to sociodemographic characteristics. The development of the instrument went through the following phases:

**Phase 1: Literature Review and Theoretical Framework**

A comprehensive literature review was conducted to establish the theoretical foundation for the assessment tool. Key databases including PubMed, Cochrane Library, and Web of Science were searched using terms related to orthodontic competency, multidisciplinary dental care, and professional assessment. The review identified four core domains essential for comprehensive orthodontic care: (1) Dental Domain: Encompassing caries risk assessment, demineralization prevention, and oral hygiene education. (2) Periodontal Domain: Including periodontal health evaluation, disease prevention, and maintenance protocols. (3) Temporomandibular Joint (TMJ) Domain: Covering temporalmandibular disorder (TMD) recognition, management strategies, and patient education. (4) Myofunctional Domain: Addressing oral habits, muscle function training, and breathing pattern disorders

**Phase 2: Expert Consultation and Initial Framework Development (Round 1 review)**

A panel of five experienced orthodontists, including one of the authors (H.A.) with over 30 years of clinical experience, participated in structured discussions to develop the initial assessment framework. The expert panel reviewed current orthodontic practice guidelines and identified key competency areas within each domain.The panel established three complementary assessment approaches: (1) Subjective competency assessment: Self-reported confidence and perceived competence. (2) Clinical practice behavior assessment: Actual implementation of multidisciplinary approaches in practice. (3) Objective knowledge testing: Evidence-based knowledge across the four domains.

**Phase 3: Multidisciplinary Expert Review (Round 2 review)**

To ensure content validity and clinical relevance, the initial framework was reviewed by a multidisciplinary panel consisting of: 3 orthodontists with subspecialty expertise, 2 periodontists with experience in orthodontic-periodontal interactions, 2 oral and maxillofacial surgeons familiar with TMJ disorders, 1 temporomandibular joint specialist. Each expert reviewed the proposed competency domains and assessment items for clinical relevance and importance,clarity of language and terminology and comprehensiveness of coverage within each domain. Expert feedback was systematically collected and incorporated into the instrument refinement process.

**Phase 4: Instrument Development**

### Subjective Competency Scale (SCS)

The SCS was designed to assess orthodontists' self-perceived competence across the four domains. Items were developed to evaluate confidence levels in: Recognition and diagnosis of conditions within each domain, implementation of preventive and treatment strategies and patient education and counseling abilities. Each item uses a 5-point Likert scale ranging from 1 (Very poor) to 5 (Excellent).

### Clinical Practice Behavior Scale (CPBS)

### The CPBS measures actual clinical behaviors and practices implemented by orthodontists. Items assess the frequency of specific clinical actions across the treatment continuum: Pre-treatment assessment and risk evaluation, Intra-treatment monitoring and intervention and post-treatment maintenance and follow-up. Items are rated on a 5-point frequency scale from 1 (never) to 5 (always).

### Objective Knowledge Test for Orthodontists (OKTO)

### The OKTO consists of 19 multiple-choice questions designed to assess evidence-based knowledge across the four competency domains. Questions were developed based on current clinical practice guidelines and recent scientific literature. Each domain is represented by 4-5 questions covering: Dental Domain (Questions C1-C5): Caries risk assessment, demineralization detection, prevention strategies, oral hygiene techniques, and fluoride application protocols. Periodontal Domain (Questions C6-C10): Periodontal health requirements, treatment modifications, maintenance schedules, and periodontal maintenance tools. TMJ Domain (Questions C11-C15): TMD examination procedures, diagnostic imaging interpretation, treatment principles, and patient education. Myofunctional Domain (Questions C16-C19): Muscle training exercises, airway assessment, habit correction appliances, and referral protocols.

### Two additional questions (Questions D1-D2) were included as attention checks to ensure respondent engagement and verify basic orthodontic knowledge.

### **Phase 5: Scoring and Composite Index Development**

### A composite Multidisciplinary Treatment (MDT) competency score was developed using weighted contributions from each component:

### MDT Score = 0.3 × SCS + 0.4 × CPBS + 0.3 × OKTO

### SCS and CPBS: Mean scores calculated across items within each scale (range 1-5), OKTO: Total correct responses (range 0-19).

The weighting scheme prioritizes actual clinical behavior (CPBS) while maintaining balanced representation of perceived competency and objective knowledge, following Miller's pyramid of clinical competence which emphasizes "does" over "knows".

**Quality Assurance Measures**

### Content validity was established through expert review, with items included only after achieving consensus among the multidisciplinary panel.Two fundamental orthodontic knowledge questions were strategically placed to identify inattentive respondents and ensure data quality. Responses with incorrect answers to these basic questions were excluded from analysis. Before formal survey distribution, the instrument was pilot tested with a group of orthodontic residents in an academic dental center in China to identify any ambiguities in question wording or technical issues with the online survey platform. A subset of 35 respondents were retested after an interval of 2 to 4 weeks to assess the temporal stability of the instrument.
