# Supplementary material for: Development and application of a multidimensional instrument to evaluate competency discrepancies in orthodontic practice
Source: BMC Oral Health. 2026 Apr 6;26:775. doi: 10.1186/s12903-026-08237-2 (PMC13134201; doi:10.1186/s12903-026-08237-2)
Supplement: Supplementary file 2 — Supplementary Material 2. [file 12903_2026_8237_MOESM2_ESM.docx]

**Supplementary File 2. Questionnaire**

**Part A: Subjective Competency Scale (SCS)**

1. **Please rate your knowledge level for the following items:**

| **A1. Recognition of dental caries and tooth demineralization** | A. Excellent | B. Good | C. Average | D. Poor | E. Very poor |
| --- | --- | --- | --- | --- | --- |
| **A2. Prevention and intervention of tooth demineralization during orthodontic treatment** | A. Excellent | B. Good | C. Average | D. Poor | E. Very poor |
| **A3. Health education on dental hygiene for patients** | A. Excellent | B. Good | C. Average | D. Poor | E. Very poor |
| **A4. Recognition of periodontal disease** | A. Excellent | B. Good | C. Average | D. Poor | E. Very poor |
| **A5. Prevention and intervention of periodontal disease during orthodontic treatment** | A. Excellent | B. Good | C. Average | D. Poor | E. Very poor |
| **A6. Periodontal health maintenance education for patients** | A. Excellent | B. Good | C. Average | D. Poor | E. Very poor |
| **A7. Recognition of temporomandibular joint disorders** | A. Excellent | B. Good | C. Average | D. Poor | E. Very poor |
| **A8. Treatment strategies for patients with temporomandibular joint disorders** | A. Excellent | B. Good | C. Average | D. Poor | E. Very poor |
| **A9. Temporomandibular joint health maintenance education for patients** | A. Excellent | B. Good | C. Average | D. Poor | E. Very poor |
| **A10. Clinical recognition and correction of bad oral habits** | A. Excellent | B. Good | C. Average | D. Poor | E. Very poor |
| **A11. Application of myofunctional training in Orthodontic Treatment** | A. Excellent | B. Good | C. Average | D. Poor | E. Very poor |
| **A12. Treatment strategies for mouth Breathing** | A. Excellent | B. Good | C. Average | D. Poor | E. Very poor |

**Part B:Clinical Practice Behavior Scale (CPBS)**

1. **Please select the frequency of your clinical practices.**

**B1. Pre-treatment examination of patient's crown integrity**

A. Never

B. Rarely (less than 50% of patients)

C. Sometimes (approximately 50% of patients)

D. Often (more than 50% of patients)

E. Always (all patients)

**B2. Pre-treatment radiographic examination of patient's caries status**

A. Never

B. Rarely (less than 50% of patients)

C. Sometimes (approximately 50% of patients)

D. Often (more than 50% of patients)

E. Always (all patients)

**B3. Pre-treatment examination of patient's periodontal probing depth and bleeding points**

A. Never

B. Rarely (less than 50% of patients)

C. Sometimes (approximately 50% of patients)

D. Often (more than 50% of patients)

E. Always (all patients)

**B4. Pre-treatment radiographic examination of patient's alveolar bone width and height**

A. Never

B. Rarely (less than 50% of patients)

C. Sometimes (approximately 50% of patients)

D. Often (more than 50% of patients)

E. Always (all patients)

**B5. Pre-treatment examination for temporomandibular joint condition of patients**

A. Never

B. Rarely (less than 50% of patients)

C. Sometimes (approximately 50% of patients)

D. Often (more than 50% of patients)

E. Always (all patients)

**B6. Pre-treatment assessment of oral habits in patients**

A. Never

B. Rarely (less than 50% of patients)

C. Sometimes (approximately 50% of patients)

D. Often (more than 50% of patients)

E. Always (all patients)

**B7. Application of fluoride preparations for patients during treatment**

A. Never

B. Rarely (less than 50% of patients)

C. Sometimes (approximately 50% of patients)

D. Often (more than 50% of patients)

E. Always (all patients)

**B8. Regular supragingival scaling scheduled for patients during treatment**

A. Never

B. Rarely (less than 50% of patients)

C. Sometimes (approximately 50% of patients)

D. Often (more than 50% of patients)

E. Always (all patients)

**B9. Regular examination of patient's temporomandibular joint condition during treatment**

A. Never

B. Rarely (less than 50% of patients)

C. Sometimes (approximately 50% of patients)

D. Often (more than 50% of patients)

E. Always (all patients)

**B10. Patient education on myofunctional training during treatment**

A. Never

B. Rarely (less than 50% of patients)

C. Sometimes (approximately 50% of patients)

D. Often (more than 50% of patients)

E. Always (all patients)

**B11. Post-treatment patient education on proper brushing techniques and other health education behaviors**

A. Never

B. Rarely (less than 50% of patients)

C. Sometimes (approximately 50% of patients)

D. Often (more than 50% of patients)

E. Always (all patients)

**B12. Post-treatment patient education on the importance of regular supragingival scaling**

A. Never

B. Rarely (less than 50% of patients)

C. Sometimes (approximately 50% of patients)

D. Often (more than 50% of patients)

E. Always (all patients)

**B13. Post-treatment patient education on temporomandibular joint health maintenance**

A. Never

B. Rarely (less than 50% of patients)

C. Sometimes (approximately 50% of patients)

D. Often (more than 50% of patients)

E. Always (all patients)

**B14. Post-treatment instruction for patients to perform myofunctional training to maintain treatment outcomes**

A. Never

B. Rarely (less than 50% of patients)

C. Sometimes (approximately 50% of patients)

D. Often (more than 50% of patients)

E. Always (all patients)

**Part C: Objective Knowledge Test for Orthodontists (OKTO).**

**Please choose the correct answer. (Single choice question)**

Note: In this presentation, correct answers are indicated in bold type.

**C1. Which of the following is the most important clinical disease indicator when evaluating enamel demineralization risk in adults?**

1. Salivary secretion and buffering capacity
2. Patient's age

**C. New cavities or dentinal lesions**

D. Dietary habits and sugar intake

**C2. Which of the following is the most appropriate clinical examination method for early diagnosis of enamel demineralization (white spot lesions) during orthodontic treatment?**

A. Using laser fluorescence detectors for diagnosis

B. Probing tooth surface texture and morphology

**C. Visual examination under bright light after drying the tooth surface for 5 seconds**

D. Radiographic examination for early demineralization lesions

**C3. During orthodontic treatment, which operation is most likely to cause enamel demineralization?**

A. Acid etching for less than 15 seconds

**B. Reducing polishing procedures after interproximal reduction**

C. Removal of excess bonding agent and resin

D. Fluoride application after interproximal reduction

**C4. Which of the following is NOT a core principle of the modified Bass brushing technique?**

A. Avoiding hard-bristled toothbrushes

B. Short horizontal vibratory movements

C. Sweeping motions

**D. Horizontal scrubbing**

**C5. Regarding the use of fluoride preparations, which statement is correct?**

A. Patients should rinse immediately after fluoride application

B. Isolation is not required during fluoride application

**C. Patients with enamel hypoplasia can receive enhanced fluoride application**

D. Liquid foods can be consumed within 30 minutes after fluoride application

**C6. What is the minimum periodontal probing requirement before orthodontic treatment in patients with periodontal disease?**

1. No pockets ≥3mm with BOP (Bleeding on Probing)

**B. No pockets ≥5mm with BOP**
C. No pockets ≥6mm with BOP

D. No pockets ≥7mm with BOP

**C7. Which of the following is NOT recommended during orthodontic treatment in patients with periodontal disease?**

**A. Patients with severe periodontitis should prioritize using clear aligners**

B. Wire ligation is preferred over elastic ties to reduce plaque accumulation

C. Use light and continuous orthodontic forces

D. Delay bonding brackets on teeth that will not be adjusted immediately

**C8. Which of the following statements about the frequency of supragingival scaling is correct?**

A. Periodontally healthy patients: every 2 years

**B. Patients with moderate to severe periodontitis: every 3 months**

C. Children undergoing orthodontic treatment: every month

D. Patients with peri-implant mucositis: every month

**C9. Which of the following is NOT a periodontal maintenance tool for orthodontic patients?**

A. Water flosser

B. Dental floss

C. Interdental brush

**D. Orthodontic wax**

**C10. What is the first-line treatment for gingival hyperplasia in orthodontic patients?**

A. Immediately stop orthodontic treatment

**B. Enhanced oral hygiene instruction + supragingival scaling**

C. Topical antibiotic ointment application

D. Gingivectomy

**C11. Which of the following is NOT part of the routine** **examination for temporomandibular joint disorders?**

A. Mouth opening range and pattern examination

B. Psychological status assessment

C. Masticatory muscle palpation

**D. Lateral cephalometric radiograph**

**C12. Anterior joint space widening, posterior joint space narrowing, with intact condylar cortex. Which CBCT image corresponds to this description?**

A.
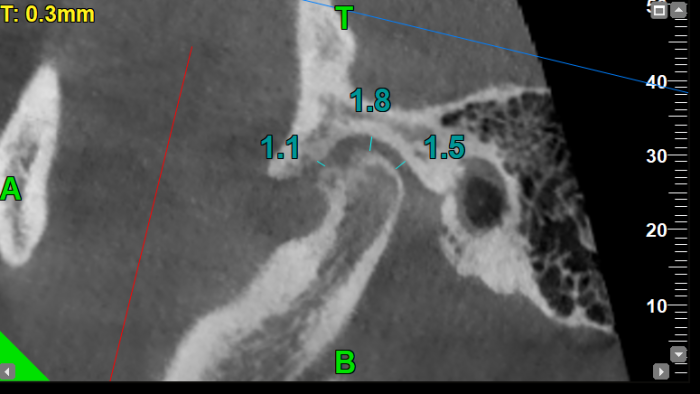
 **B.**
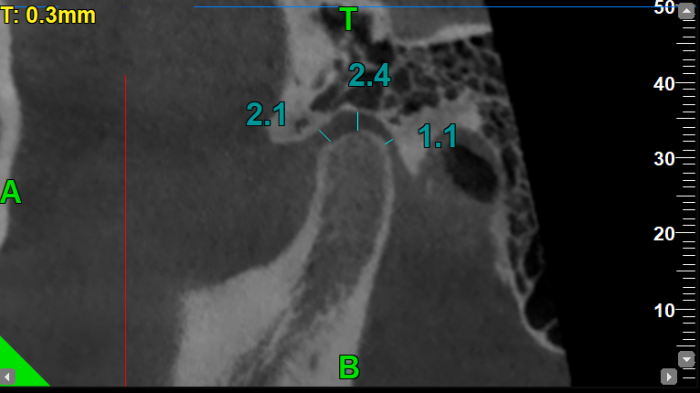


C.
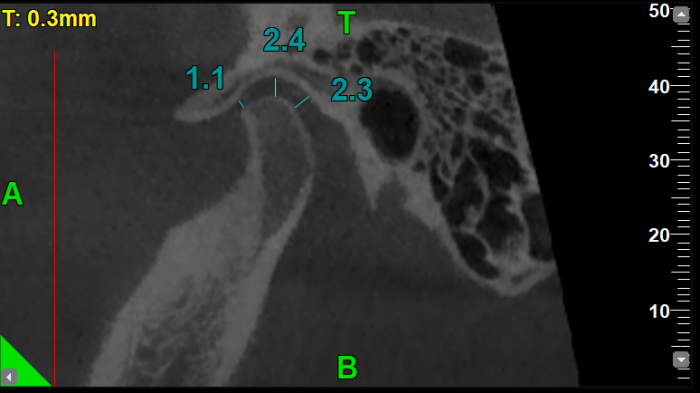
 D.
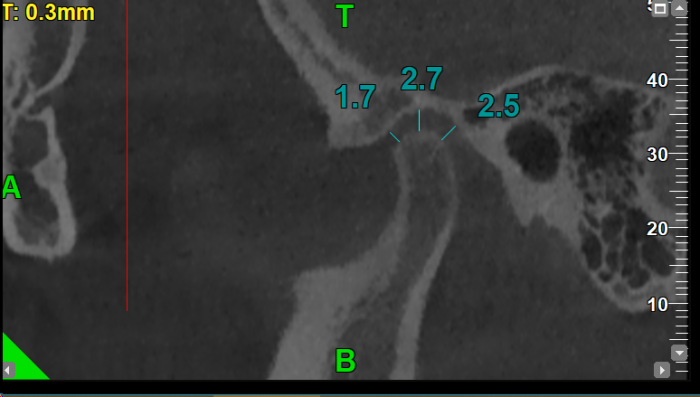


**C13. Which statement most accurately describes the relationship between orthodontic treatment and temporomandibular disorders (TMD)?**

A. Orthodontic treatment can prevent most TMD occurrences

**B. There is no clear causal relationship between orthodontic treatment and TMD occurrence**

C. Extraction orthodontics increases TMD risk

D. Class II malocclusion inevitably leads to TMD

**C14. Which of the following is the primary treatment principle for temporomandibular disorders (TMD)?**

A. Surgery as first-line treatment
**B. Conservative treatment as first-line therapy**
C. Occlusal adjustment as first-line treatment
D. Orthodontic treatment as first-line therapy

**C15.Which of the following is INCORRECT regarding daily health maintenance for patients with temporomandibular disorders (TMD)?**

A. Avoid wide mouth opening

B. Heat application to the joint area can relieve muscle pain

**C. Keep teeth tightly clenched to stabilize the joint**

D. Improve sleep quality and maintain a pleasant mood

**C16. Which of the following myofunctional training exercises is INCORRECTLY matched?**

A. Tongue tip elevation exercises — Lingual muscle function

C. Tongue-lip coordination exercises — Swallowing function

B. Cheek puffing exercises — Buccal muscle function

**D. Lower lip biting exercises — Orbicularis oris muscle function**

**C17. In cephalometric analysis, high OSA risk is indicated when posterior airway space (PAS) is less than:**

**A.11mm**

B.6-10 mm

C. 5mm

D. 3mm

**C18.Which appliance-habit pairing is INCORRECT?**

A. Tongue thrusting habit — Tongue crib

B. Mouth breathing — Vestibular screen

C. Digit sucking habit — Palatal crib

**D. Unilateral chewing — Lip bumper**

**C19.A 10-year-old child presents with nocturnal mouth breathing and snoring for 6 months. Physical examination reveals Grade III adenoid hypertrophy. What is the most appropriate initial management?**

A. Rapid maxillary expansion

**B. Ear, Nose, and Throat（ENT）referral for evaluation**

C. Vestibular screen appliance therapy

D. Orofacial myofunctional therapy

(Ballikaya et al. 2018; Lin et al. 2022; Alzahrani et al. 2023; Kandasamy 2025)

**Note:**

The following two questions were used to assess the seriousness of respondents' participation and test basic orthodontic knowledge.

**D1. Which of the following is NOT part of Angle's classification?**

A. Angle Class II

B. Angle Class II Division I

C. Angle Class II Division II

**D. Angle Class IV**

**D2. Which of the following is NOT a commonly used measurement in cephalometric analysis?**

A.SNA

B.U1-L1

**C.SUV**

D.ANB

**Reference**

1. Ahovuo-Saloranta A, Forss H, Hiiri A, Nordblad A, Mäkelä M. 2016. Pit and fissure sealants versus fluoride varnishes for preventing dental decay in the permanent teeth of children and adolescents. Cochrane Database Syst Rev. 2016(1):CD003067. doi:10.1002/14651858.CD003067.pub4.
2. Alzahrani HA, Alkhaldi RO, Alsufyani DH, Alghamdi SA, Althobity TA, Fageeh YA. 2023. Adenoid Hypertrophy and Orthodontic Complications: An Assessment of Parental Knowledge in Saudi Arabia. Cureus. 15(7):e41692. doi:10.7759/cureus.41692.
3. Baik A, Alamoudi N, El-Housseiny A, Altuwirqi A. 2021. Fluoride Varnishes for Preventing Occlusal Dental Caries: A Review. Dent J (Basel). 9(6):64. doi:10.3390/dj9060064.
4. Ballikaya E, Guciz Dogan B, Onay O, Uzamis Tekcicek M. 2018. Oral health status of children with mouth breathing due to adenotonsillar hypertrophy. Int J Pediatr Otorhinolaryngol. 113:11–15. doi:10.1016/j.ijporl.2018.07.018.
5. Boyd K, Saccomanno S, Lewis CJ, Coceani Paskay L, Quinzi V, Marzo G. 2021. Myofunctional therapy. Part 1: Culture, industrialisation and the shrinking human face. Eur J Paediatr Dent. 22(1):80–81. doi:10.23804/ejpd.2021.22.01.15.
6. Coronel-Zubiate F-T, Marroquín-Soto C, Geraldo-Campos L-A, Aguirre-Ipenza R, Urbano-Rosales L-M, Luján-Valencia S-A, Tozo-Burgos J-G, Arbildo-Vega H-I. 2022. Association between orthodontic treatment and the occurrence of temporomandibular disorders: A systematic review and meta-analysis. J Clin Exp Dent. 14(12):e1032–e1043. doi:10.4317/jced.59970.
7. Danesh G, Hellak A, Lippold C, Ziebura T, Schafer E. 2007. Enamel surfaces following interproximal reduction with different methods. Angle Orthod. 77(6):1004–1010. doi:10.2319/041806-165.1.
8. Danesh G, Podstawa PKK, Schwartz C-E, Kirschneck C, Bizhang M, Arnold WH. 2020. Depth of acid penetration and enamel surface roughness associated with different methods of interproximal enamel reduction. PLoS One. 15(3):e0229595. doi:10.1371/journal.pone.0229595.
9. Enamel demineralization after different methods of interproximal polishing - PubMed. [accessed 2025 Aug 18]. https://pubmed.ncbi.nlm.nih.gov/25689983/.
10. Farooqi OA, Wehler CJ, Gibson G, Jurasic MM, Jones JA. 2015. Appropriate Recall Interval for Periodontal Maintenance: A Systematic Review. J Evid Based Dent Pract. 15(4):171–181. doi:10.1016/j.jebdp.2015.10.001.
11. Featherstone JDB, Chaffee BW. 2018. The Evidence for Caries Management by Risk Assessment (CAMBRA®). Adv Dent Res. 29(1):9–14. doi:10.1177/0022034517736500.
12. Featherstone JDB, Crystal YO, Alston P, Chaffee BW, Doméjean S, Rechmann P, Zhan L, Ramos-Gomez F. 2021a. Evidence-Based Caries Management for All Ages-Practical Guidelines. Front Oral Health. 2:657518. doi:10.3389/froh.2021.657518.
13. Featherstone JDB, Crystal YO, Alston P, Chaffee BW, Doméjean S, Rechmann P, Zhan L, Ramos-Gomez F. 2021b. A Comparison of Four Caries Risk Assessment Methods. Front Oral Health. 2:656558. doi:10.3389/froh.2021.656558.
14. Ferreira Zandoná AG, Analoui M, Beiswanger BB, Isaacs RL, Kafrawy AH, Eckert GJ, Stookey GK. 1998. An in vitro comparison between laser fluorescence and visual examination for detection of demineralization in occlusal pits and fissures. Caries Res. 32(3):210–218. doi:10.1159/000016455.
15. Ferrillo M, Giudice A, Marotta N, Fortunato F, Di Venere D, Ammendolia A, Fiore P, de Sire A. 2022. Pain Management and Rehabilitation for Central Sensitization in Temporomandibular Disorders: A Comprehensive Review. Int J Mol Sci. 23(20):12164. doi:10.3390/ijms232012164.
16. Janakiram C, Varghese N, Venkitachalam R, Joseph J, Vineetha K. 2020. Comparison of modified Bass, Fones and normal tooth brushing technique for the efficacy of plaque control in young adults- A randomized clinical trial. J Clin Exp Dent. 12(2):e123–e129. doi:10.4317/jced.55747.
17. Kandasamy S. 2025. Mouth breathing and orthodontic intervention: Does the evidence support keeping our mouths shut? Am J Orthod Dentofacial Orthop. 167(6):629–634. doi:10.1016/j.ajodo.2025.02.005.
18. Kwon T, Lamster IB, Levin L. 2021. Current Concepts in the Management of Periodontitis. Int Dent J. 71(6):462–476. doi:10.1111/idj.12630.
19. Lin L, Zhao T, Qin D, Hua F, He H. 2022. The impact of mouth breathing on dentofacial development: A concise review. Front Public Health. 10:929165. doi:10.3389/fpubh.2022.929165.
20. Saba ES, Kim H, Huynh P, Jiang N. 2024. Orofacial Myofunctional Therapy for Obstructive Sleep Apnea: A Systematic Review and Meta-Analysis. Laryngoscope. 134(1):480–495. doi:10.1002/lary.30974.
21. Shaffer SM, Brismée J-M, Sizer PS, Courtney CA. 2014. Temporomandibular disorders. Part 2: conservative management. J Man Manip Ther. 22(1):13–23. doi:10.1179/2042618613Y.0000000061.
22. Shick RA. 1981. Maintenance phase of periodontal therapy. J Periodontol. 52(9):576–583. doi:10.1902/jop.1981.52.9.576.
23. Zhong W, Zhou C, Yin Y, Feng G, Zhao Z, Pan Y, Bai Y, Jin Z, Xu Y, Fang B, et al. 2025. Expert consensus on orthodontic treatment of patients with periodontal disease. Int J Oral Sci. 17(1):27. doi:10.1038/s41368-025-00356-w.
