# Supplementary material for: Development and application of a multidimensional instrument to evaluate competency discrepancies in orthodontic practice
Source: BMC Oral Health. 2026 Apr 6;26:775. doi: 10.1186/s12903-026-08237-2 (PMC13134201; doi:10.1186/s12903-026-08237-2)
Supplement: Supplementary file 3 — Supplementary Material 3. [file 12903_2026_8237_MOESM3_ESM.docx]

**Supplementary File 3. Figures**

**Supplementary File 3 Figures 1. Responses to Subjective Competency Scale (SCS)**


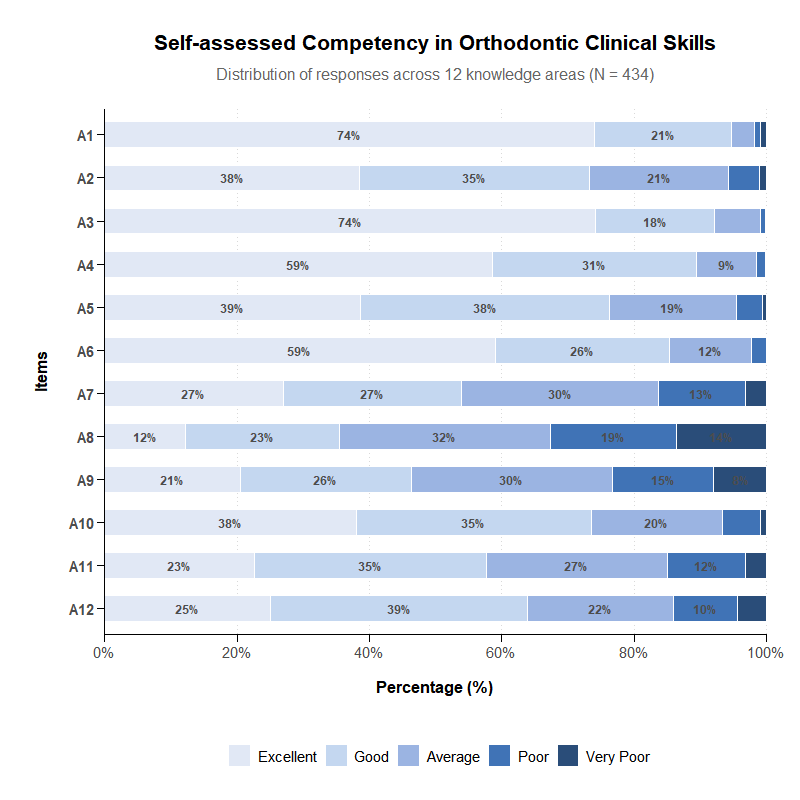


Note: Items included: A1. Recognition of dental caries and tooth demineralization; A2. Prevention and intervention of tooth demineralization during orthodontic treatment; A3. Health education on dental hygiene for patients; A4. Recognition of periodontal disease; A5. Prevention and intervention of periodontal disease during orthodontic treatment; A6. Periodontal health maintenance education for patients; A7. Recognition of temporomandibular joint disorders; A8. Treatment strategies for patients with temporomandibular joint disorders; A9. Temporomandibular joint health maintenance education for patients; A10. Clinical recognition and correction of bad oral habits; A11. Application of myofunctional training in Orthodontic Treatment; A12. Treatment strategies for mouth Breathing

**Supplementary File 3 Figures 2. Responses to Clinical Practice Behavior Scale (CPBS)**


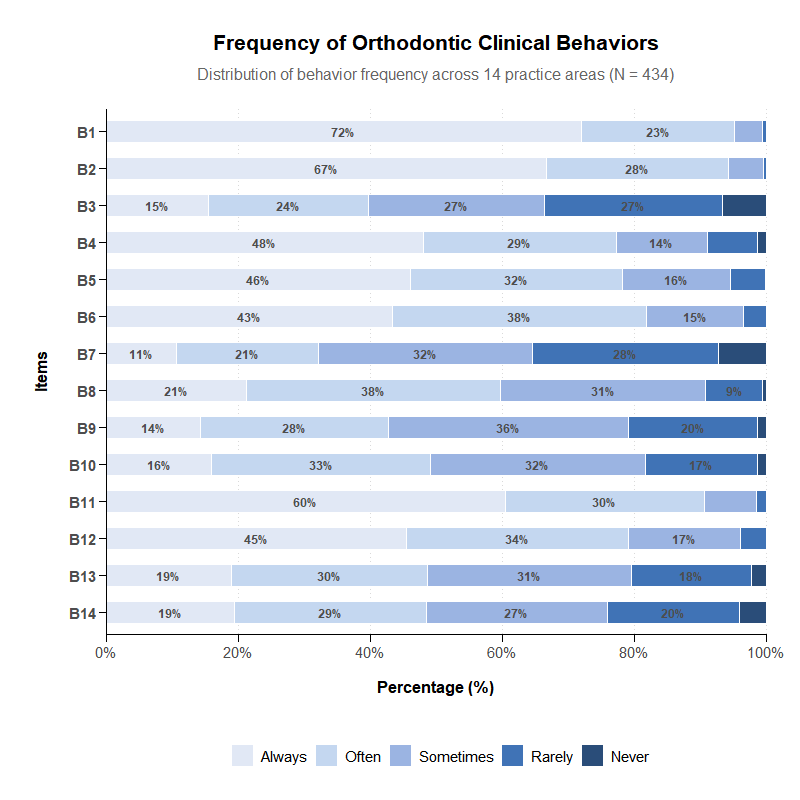


- Note: Items included: B1. Pre-treatment examination of patient's crown integrity; B2. Pre-treatment radiographic examination of patient's caries status; B3. Pre-treatment examination of patient's periodontal probing depth and bleeding points; B4. Pre-treatment radiographic examination of patient's alveolar bone width and height; B5. Pre-treatment examination for temporomandibular joint condition of patients; B6. Pre-treatment assessment of oral habits in patients; B7. Application of fluoride preparations for patients during treatment; B8. Regular supragingival scaling scheduled for patients during treatment; B9. Regular examination of patient's temporomandibular joint condition during treatment; B10. Patient education on myofunctional training during treatment; B11. Post-treatment patient education on proper brushing techniques and other health education behaviors; B12. Post-treatment patient education on the importance of regular supragingival scaling; B13. Post-treatment patient education on temporomandibular joint health maintenance; B14. Post-treatment instruction for patients to perform myofunctional training to maintain treatment outcomes

**Supplementary File 3 Figures 3. Comparison of Domain-Specific Scores Across Three Assessment Scales**

**
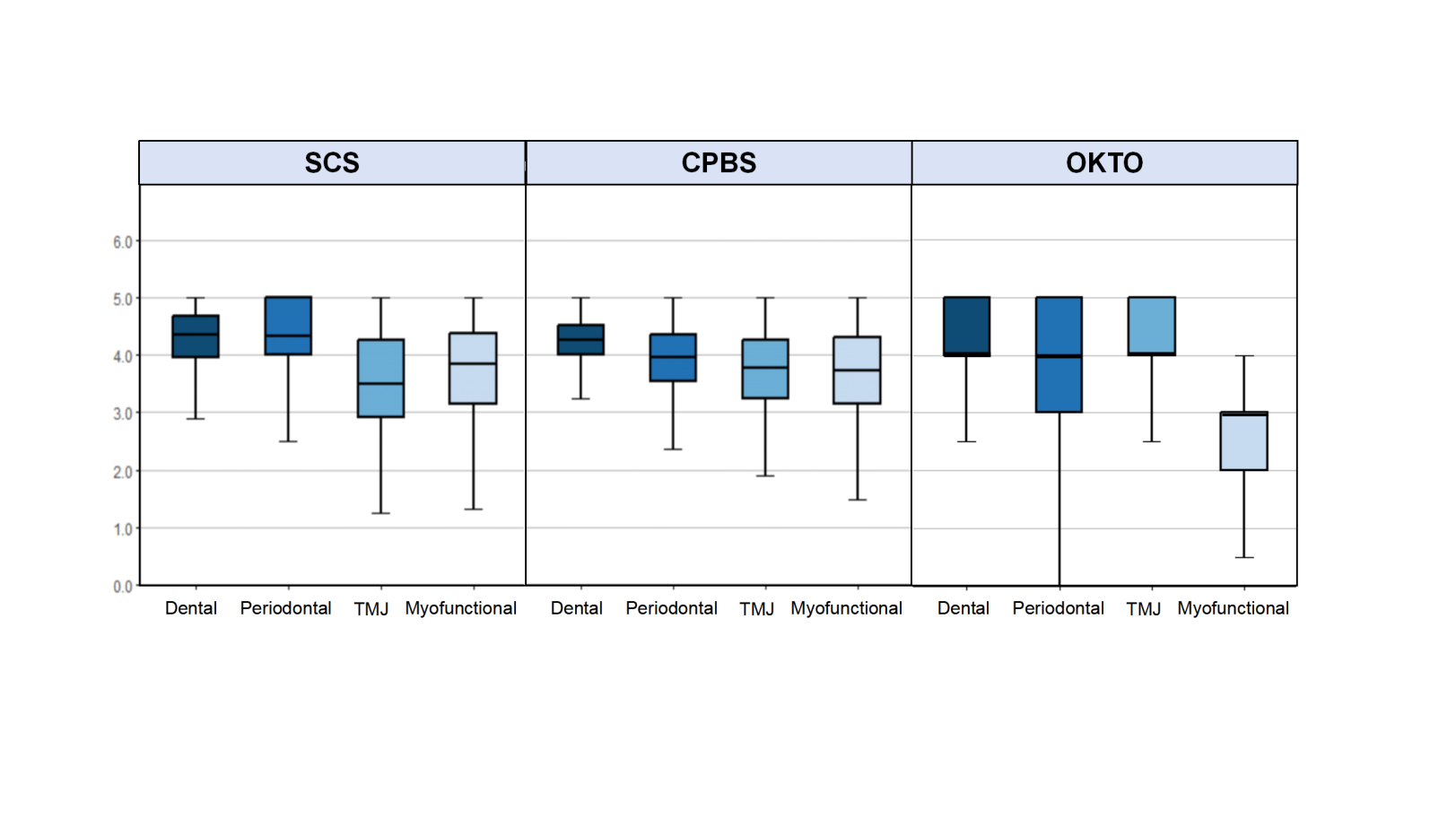
**

Note: SCS, Subjective Competency Scale; CPBS, Clinical Practice Behavior Scale; OKTO, Objective Knowledge Test for Orthodontists. All scales showed significant differences across domains (Friedman test, all p < 0.001). Box plots display median, interquartile range, and outliers for each domain within each scale.
