# Supplementary material for: Development and application of a multidimensional instrument to evaluate competency discrepancies in orthodontic practice
Source: BMC Oral Health. 2026 Apr 6;26:775. doi: 10.1186/s12903-026-08237-2 (PMC13134201; doi:10.1186/s12903-026-08237-2)
Supplement: Supplementary file 4 — Supplementary Material 4. [file 12903_2026_8237_MOESM4_ESM.docx]

**Supplementary File 4. Tables**

**Supplementary File 4 Table 1. Factor Analysis Results for Subjective Competency Scale (SCS)**

|  | **Factor loading*** | | | |  |
| --- | --- | --- | --- | --- | --- |
| **Item** | **1** | **2** | **3** | **4** | **Item - Rest Correlation** |
| A1. Recognition of dental caries and tooth demineralization | 0.009 | 0.182 | 0.091 | **0.88** | 0.369 |
| A2. Prevention and intervention of tooth demineralization during orthodontic treatment | 0.232 | 0.325 | 0.206 | **0.685** | 0.564 |
| A3. Health education on dental hygiene for patients | -0.112 | **0.403** | 0.306 | **0.737** | 0.429 |
| A4. Recognition of periodontal disease | 0.198 | **0.699** | 0.036 | 0.303 | 0.503 |
| A5. Prevention and intervention of periodontal disease during orthodontic treatment | 0.355 | **0.660** | 0.074 | 0.35 | 0.615 |
| A6. Periodontal health maintenance education for patients | 0.179 | **0.859** | 0.16 | 0.072 | 0.562 |
| A7. Recognition of temporomandibular joint disorders | **0.766** | 0.164 | 0.4 | 0.127 | 0.737 |
| A8. Treatment strategies for patients with temporomandibular joint disorders | **0.868** | 0.079 | 0.293 | 0.149 | 0.698 |
| A9. Temporomandibular joint health maintenance education for patients | **0.812** | 0.195 | 0.332 | 0.028 | 0.703 |
| A10. Clinical recognition and correction of bad oral habits; | 0.373 | 0.259 | **0.748** | 0.042 | 0.707 |
| A11. Application of myofunctional training in Orthodontic Treatment | 0.311 | 0.159 | **0.836** | 0.133 | 0.696 |
| A12. Treatment strategies for mouth Breathing | 0.401 | 0.124 | **0.750** | 0.225 | 0.719 |

Note: Eigenvalues >1.0;KMO = 0.873 and the Bartlett’s test: χ² = 3023.250, p < 0.000, indicated that the data were appropriate for conducting factor analysis. Cronbach’s Alpha: Overall = 0.897; Demineralization and Caries = 0.662; Periodontal = 0.810; TMJ = 0.903; Muscle = 0.879; *The bold numbers represent the items that contributed primarily to the factor for that column.

**Supplementary File 4 Table 2. Confirmatory Factor Analysis Results for Subjective Competency Scale (SCS)**

| **Factor** | **Item Description** | **Standardized Loading (λ)** | **z** | **p** | **AVE** | **CR** |
| --- | --- | --- | --- | --- | --- | --- |
| Dental | A1 | 0.568 | — | — | 0.417 | 0.679 |
|  | A2 | 0.756 | 10.13 | <0.001 |  |  |
|  | A3 | 0.597 | 9.01 | <0.001 |  |  |
| Periodontal | A4 | 0.713 | — | — | 0.593 | 0.813 |
|  | A5 | 0.759 | 14.03 | <0.001 |  |  |
|  | A6 | 0.833 | 14.91 | <0.001 |  |  |
| TMJ | A7 | 0.853 | — | — | 0.759 | 0.904 |
|  | A8 | 0.892 | 23.67 | <0.001 |  |  |
|  | A9 | 0.868 | 22.76 | <0.001 |  |  |
| Myofunctional | A10 | 0.802 | — | — | 0.713 | 0.882 |
|  | A11 | 0.863 | 19.91 | <0.001 |  |  |
|  | A12 | 0.867 | 20.01 | <0.001 |  |  |

Note: '—' indicates the reference item for each factor. Model fit indices: CFI = 0.933, TLI = 0.908, GFI = 0.906, RMSEA = 0.098 (90% CI: 0.087–0.111), SRMR = 0.048. AVE, average variance extracted; CR, composite reliability; TMJ, temporomandibular joint; TMD, temporomandibular disorder.

**Supplementary File 4 Table 3. Item Analysis Results for Objective Knowledge Test in Orthodontics (OKTO)**

| **Item** | **Difficulty Index** | **Discrimination Index** | **Point  Biserial Correlation** | **KR-20 Coefficient with the Item Omitted** |
| --- | --- | --- | --- | --- |
| C1 | 0.95 | 0.07 | 0.20 | 0.45 |
| C2 | 0.64 | 0.28 | 0.32 | 0.45 |
| C3 | 0.88 | 0.19 | 0.32 | 0.44 |
| C4 | 0.87 | 0.16 | 0.23 | 0.46 |
| C5 | 0.79 | 0.19 | 0.23 | 0.47 |
| C6 | 0.68 | 0.30 | 0.34 | 0.45 |
| C7 | 0.49 | 0.34 | 0.32 | 0.46 |
| C8 | 0.81 | 0.25 | 0.31 | 0.45 |
| C9 | 0.96 | 0.05 | 0.21 | 0.45 |
| C10 | 0.97 | 0.06 | 0.24 | 0.45 |
| C11 | 0.66 | 0.45 | 0.47 | 0.41 |
| C12 | 0.84 | 0.25 | 0.34 | 0.44 |
| C13 | 0.74 | 0.46 | 0.51 | 0.39 |
| C14 | 0.92 | 0.15 | 0.28 | 0.44 |
| C15 | 0.97 | 0.07 | 0.24 | 0.45 |
| C16 | 0.63 | 0.35 | 0.37 | 0.44 |
| C17 | 0.26 | 0.29 | 0.33 | 0.45 |
| C18 | 0.88 | 0.21 | 0.33 | 0.44 |
| C19 | 0.96 | 0.06 | 0.20 | 0.43 |

Note: Complete item content is provided in Appendix 2 (Part C).

**Supplementary File 4 Table 4. Comparison of SCS and OKTO scores across different CPBS Groups**

|  | **Median (Interquartile Range)** | | | **p^a^** | **p^b^** | **p^c^** | **p^d^** |
| --- | --- | --- | --- | --- | --- | --- | --- |
|  | **Low CPBS group**  **(n=127)** | **Middle CPBS group**  **(n=175)** | **High CPBS**  **Group**  **(n=132)** |  |  |  |  |
| **SCS** | 42(37-47) | 48(44-53) | 53(49-58) | 0.000** | 0.000** | 0.000** | 0.000** |
| **OKTO** | 17(16-18.0) | 17(16-19) | 17(15-18) | 0.017* | 0.882 | 0.296 | 0.013* |

Note: p^a^ = Kruskal-Wallis test; p^b^-p^d^ = Dunn's pairwise comparisons (Low vs Middle, Low vs High, Middle vs High respectively). CPBS,Clinical Practice Behavior Scale; SCS, Subjective Competency Scale; OKTO, Objective Knowledge Test for Orthodontists. *p < 0.05, **p < 0.01.

**Supplementary File 4 Table 5. Descriptive Statistics of Subjective Competency Scale**

| **Category** | **Item** | **Mean ± SD** | **Median (IQR)** |
| --- | --- | --- | --- |
| **Dental** |  | **4.45 ± 0.77** | **5** |
|  | A1. Recognition of dental caries and tooth demineralization | 4.66 ± 0.68 | 5 |
|  | A2. Prevention and intervention of tooth demineralization during orthodontic treatment | 4.05 ± 0.94 | 4 |
|  | A3. Health education on dental hygiene for patients | 4.65 ± 0.66 | 5 |
| **Periodontal** |  | **4.33 ± 0.81** | **5** |
|  | A4. Recognition of periodontal disease | 4.46 ± 0.74 | 5 |
|  | A5. Prevention and intervention of periodontal disease during orthodontic treatment | 4.10 ± 0.89 | 4 |
|  | A6. Periodontal health maintenance education for patients | 4.42 ± 0.80 | 5 |
| **TMJ** |  | **3.33 ± 1.17** | **3** |
|  | A7. Recognition of temporomandibular joint disorders | 3.61 ± 1.11 | 4 |
|  | A8. Treatment strategies for patients with temporomandibular joint disorders | 3.01 ± 1.21 | 3 |
|  | A9. Temporomandibular joint health maintenance education for patients | 3.36 ± 1.20 | 3 |
| **Myofunctional** |  | **3.79 ± 1.03** | **4** |
|  | A10. Clinical recognition and correction of bad oral habits; | 4.04 ± 0.95 | 4 |
|  | A11. Application of myofunctional training in Orthodontic Treatment | 3.62 ± 1.06 | 4 |
|  | A12. Treatment strategies for mouth Breathing | 3.71 ± 1.08 | 4 |

Note: Each subscale score was calculated as the mean of its constituent items. SD, standard deviation; IQR, interquartile range; TMJ, Temporomandibular Joint.

**Supplementary File 4 Table 5. Clinical Practice Behavior Frequency Distribution [n(%)]**

| **Category** | **Item** | **Never (A)** | **Rarely (B)** | **Sometimes (C)** | **Often (D)** | **Always (E)** | **Average Compliance Rate (D+E)** |
| --- | --- | --- | --- | --- | --- | --- | --- |
| **Dental** |  |  |  |  |  |  | **78.00%*** |
|  | B1 | 0 (0%) | 3 (0.69%) | 18 (4.15%) | 101 (23.27%) | 312 (71.89%) | 95.16% |
|  | B2 | 0 (0%) | 2 (0.46%) | 23 (5.30%) | 120 (27.65%) | 289 (66.59%) | 94.24% |
|  | B7 | 32 (7.37%) | 122 (28.11%) | 141 (32.49%) | 93 (21.43%) | 46 (10.60%) | 32.03% |
|  | B11 | 0 (0%) | 7 (1.61%) | 34 (7.83%) | 131 (30.18%) | 262 (60.37%) | 90.55% |
| **Periodontal** |  |  |  |  |  |  | **63.88%*** |
|  | B3 | 29 (6.68%) | 117 (26.96%) | 116 (26.73%) | 105 (24.19%) | 67 (15.44%) | 39.63% |
|  | B4 | 6 (1.38%) | 33 (7.60%) | 60 (13.82%) | 127 (29.26%) | 208 (47.93%) | 77.19% |
|  | B8 | 3 (0.69%) | 37 (8.53%) | 135 (31.11%) | 167 (38.48%) | 92 (21.20%) | 59.68% |
|  | B12 | 0 (0%) | 17 (3.92%) | 74 (17.05%) | 146 (33.64%) | 197 (45.39%) | 79.03% |
| **TMJ** |  |  |  |  |  |  | 56.45% |
|  | B5 | 1 (0.23%) | 23 (5.30%) | 71 (16.36%) | 139 (32.03%) | 200 (46.08%) | 78.11% |
|  | B9 | 6 (1.38%) | 85 (19.59%) | 158 (36.41%) | 123 (28.34%) | 62 (14.29%) | 42.63% |
|  | B13 | 10 (2.30%) | 79 (18.20%) | 134 (30.88%) | 129 (29.72%) | 82 (18.89%) | 48.61% |
| **Myofunctional** |  |  |  |  |  |  | **59.75%*** |
|  | B6 | 0 (0%) | 15 (3.46%) | 64 (14.75%) | 167 (38.48%) | 188 (43.32%) | 81.80% |
|  | B10 | 6 (1.38%) | 74 (17.05%) | 141 (32.49%) | 144 (33.18%) | 69 (15.90%) | 49.08% |
|  | B14 | 18 (4.15%) | 87 (20.05%) | 119 (27.42%) | 126 (29.03%) | 84 (19.35%) | 48.38% |

Note: *Category Average: Mean of all Standard Compliance Rates (D+E) within a clinical domain. TMJ, Temporomandibular Joint; Note: Complete item content is provided in Appendix 2 (Part B).

**Supplementary File 4 Table 6. Correct Answer Rate of Objective Knowledge Assessment**

| **Category** | **Item** | **Correct Rate** |
| --- | --- | --- |
| **Dental** |  | **82.63%*** |
|  | C1. Which of the following is the most important clinical disease indicator when evaluating enamel demineralization risk in adults? | 95.39% |
|  | C2. Which of the following is the most appropriate clinical examination method for early diagnosis of enamel demineralization (white spot lesions) during orthodontic treatment? | 64.29% |
|  | C3. During orthodontic treatment, which operation is most likely to cause enamel demineralization? | 88.25% |
|  | C4. Which of the following is NOT a core principle of the modified Bass brushing technique? | 86.64% |
|  | C5. Regarding the use of fluoride preparations, which statement is correct? | 78.57% |
| **Periodontal** |  | **73.56%*** |
|  | C6. What is the minimum periodontal probing requirement before orthodontic treatment in patients with periodontal disease? | 68.20% |
|  | C7. Which of the following is NOT recommended during orthodontic treatment in patients with periodontal disease? | 48.62% |
|  | C8. Which of the following statements about the frequency of supragingival scaling is correct? | 81.34% |
|  | C9. Which of the following is NOT a periodontal maintenance tool for orthodontic patients? | 96.08% |
|  | C10. What is the first-line treatment for gingival hyperplasia in orthodontic patients? | 97.24% |
| **TMJ** |  | **82.58%*** |
|  | C11. Which of the following is NOT part of the routine examination for temporomandibular joint disorders? | 66.13% |
|  | C12. Anterior joint space widening, posterior joint space narrowing, with intact condylar cortex. Which CBCT image corresponds to this description? | 83.64% |
|  | C13. Which statement most accurately describes the relationship between orthodontic treatment and temporomandibular disorders (TMD)? | 73.96% |
|  | C14. Which of the following is the primary treatment principle for temporomandibular disorders (TMD)? | 91.94% |
|  | C15.Which of the following is INCORRECT regarding daily health maintenance for patients with temporomandibular disorders (TMD)? | 97.00% |
| **Myofunctional** |  | **68.26%*** |
|  | C16. Which of the following myofunctional training exercises is INCORRECTLY matched? | 62.90% |
|  | C17. In cephalometric analysis, high OSA risk is indicated when posterior airway space (PAS) is less than: | 25.58% |
|  | C18.Which appliance-habit pairing is INCORRECT? | 87.56% |
|  | C19.A 10-year-old child presents with nocturnal mouth breathing and snoring for 6 months. Physical examination reveals Grade III adenoid hypertrophy. What is the most appropriate initial management? | 96.31% |

Note: * The category average is the mean of the correct rates for all items within a specific knowledge domain. TMJ, Temporomandibular Joint.

**Supplementary File 4 Table 7. Demographic Factors and MDT Competency Levels**

| **Characteristic** | **No. (%)** | | | **χ2** | **p** |
| --- | --- | --- | --- | --- | --- |
|  | **Low MDT**  **group** | **Middle**  **MDT group** | **High**  **MDT group** |  |  |
| **Age group** |  |  |  |  |  |
| 18~25 | 14(8.38) | 1(0.74) | 1(0.76) | 41.675 | 0.000** |
| 26~30 | 26(15.57) | 20(14.71) | 12(9.16) |  |  |
| 31~40 | 78(46.71) | 71(52.21) | 56(42.75) |  |  |
| 41~50 | 39(23.35) | 30(22.06) | 38(29.01) |  |  |
| 51~60 | 10(5.99) | 14(10.29) | 17(12.98) |  |  |
| 60 | 0(0.00) | 0(0.00) | 7(5.34) |  |  |
| **Educational level** |  |  |  |  |  |
| Associate Degree | 38(22.75) | 10(7.35) | 7(5.34) | 49.064 | 0.000** |
| Bachelor’s Degree | 85(50.90) | 50(36.76) | 58(44.27) |  |  |
| Master’s Degree | 39(23.35) | 58(42.65) | 44(33.59) |  |  |
| Doctoral Degree | 5(2.99) | 18(13.24) | 22(16.79) |  |  |
| **Job Title** |  |  |  |  |  |
| Junior (resident) | 52(31.14) | 35(25.74) | 18(13.74) | 34.676 | 0.000** |
| Intermediate (attending) | 81(48.50) | 59(43.38) | 54(41.22) |  |  |
| Subsenior (associate chief) | 30(17.96) | 32(23.53) | 35(26.72) |  |  |
| Senior (chief) | 4(2.40) | 10(7.35) | 24(18.32) |  |  |
| **Years of practice in orthodontics** |  |  |  |  |  |
| ＜1 year | 30(17.96) | 19(13.97) | 10(7.63) | 32.186 | 0.000** |
| 1-3 years | 51(30.54) | 29(21.32) | 23(17.56) |  |  |
| 4-6 years | 26(15.57) | 19(13.97) | 14(10.69) |  |  |
| 7-10 years | 30(17.96) | 27(19.85) | 26(19.85) |  |  |
| 11-20 years | 18(10.78) | 28(20.59) | 31(23.66) |  |  |
| ＞20 years | 12(7.19) | 14(10.29) | 27(20.61) |  |  |
| **Workplace** |  |  |  |  |  |
| Private Dental Clinic | 70(41.92) | 30(22.06) | 36(27.48) | 24.853 | 0.016* |
| Chain Dental Institution | 32(19.16) | 25(18.38) | 24(18.32) |  |  |
| Community Hospital | 3(1.80) | 4(2.94) | 2(1.53) |  |  |
| Secondary A-level General Hospital (Dental Department) | 11(6.59) | 9(6.62) | 4(3.05) |  |  |
| Secondary A-level Dental Hospital | 5(2.99) | 4(2.94) | 8(6.11) |  |  |
| Tertiary A-level General Hospital (Dental Department) | 29(17.37) | 36(26.47) | 31(23.66) |  |  |
| Tertiary A-level Dental Hospital | 17(10.18) | 28(20.59) | 26(19.85) |  |  |
| **New orthodontic cases per year** |  |  |  |  |  |
| <20 | 69(41.32) | 37(27.21) | 35(26.72) | 38.049 | 0.000** |
| 21-50 | 49(29.34) | 28(20.59) | 22(16.79) |  |  |
| 51-100 | 34(20.36) | 26(19.12) | 30(22.90) |  |  |
| 100-200 | 10(5.99) | 27(19.85) | 27(20.61) |  |  |
| >200 | 5(2.99) | 18(13.24) | 17(12.98) |  |  |
| **Orthodontic CE courses attended (past 2 years)** |  |  |  |  |  |
| **0 times** | **8(4.79)** | **10(7.35)** | **4(3.05)** | **14.987** | **0.020*** |
| 1-3 times | 94(56.29) | 57(41.91) | 50(38.17) |  |  |
| 4-6 times | 30(17.96) | 35(25.74) | 36(27.48) |  |  |
| >6 times | 35(20.96) | 34(25.00) | 41(31.30) |  |  |

Note: **p < 0.01; *p < 0.05. MDT, Multidisciplinary Team; CE, Continuing Education. Chi-square test used for group comparisons. Secondary/Tertiary A-level hospitals represent the Chinese hospital grading system.

**Supplementary File 4 Table 8. Responses to Objective Knowledge Test for Orthodontists (OKTO)**

| **Items** | **Options** | **Response [n(%)]** |
| --- | --- | --- |
| **C1. Which of the following is the most important clinical disease indicator when evaluating enamel demineralization risk in adults?** | A.Salivary secretion and buffering capacity Patient's age | 9 (2.07%) |
|  | B. Patient's age | 3 (0.69%) |
|  | **C. New cavities or dentinal lesions** | **414 **(95.39%)**** |
|  | **D. Dietary habits and sugar intake** | **8 (1.84%)** |
| **C2. Which of the following is the most appropriate clinical examination method for early diagnosis of enamel demineralization (white spot lesions) during orthodontic treatment?** | A. Using laser fluorescence detectors for diagnosis | 92 (21.2%) |
|  | B. Probing tooth surface texture and morphology | 38 (8.76%) |
|  | ****C. Visual examination under bright light after drying the tooth surface for 5 seconds**** | ****279 (64.29%)**** |
|  | D. Radiographic examination for early demineralization lesions | 25 (5.76%) |
| **C3. During orthodontic treatment, which operation is most likely to cause enamel demineralization?** | A. Acid etching for less than 15 seconds | 11 (2.53%) |
|  | ****B. Reducing polishing procedures after interproximal reduction**** | ****383 (88.25%)**** |
|  | C. Removal of excess bonding agent and resin | 35 (8.06%) |
|  | D. Fluoride application after interproximal reduction | 5 (1.15%) |
| **C4. Which of the following is NOT a core principle of the modified Bass brushing technique?** | A. Avoiding hard-bristled toothbrushes | 32 (7.37%) |
|  | B. Short horizontal vibratory movements | 21 (4.84%) |
|  | C. Sweeping motions | 5 (1.15%) |
|  | ****D. Horizontal scrubbing**** | ****376 (86.64%)**** |
| **C5. Regarding the use of fluoride preparations, which statement is correct?** | A. Patients should rinse immediately after fluoride application | 8 (1.84%) |
|  | B. Isolation is not required during fluoride application | 24 (5.53%) |
|  | ****C. Patients with enamel hypoplasia can receive enhanced fluoride application**** | ****341 (78.57%)**** |
|  | D. Liquid foods can be consumed within 30 minutes after fluoride application | 61 (14.06%) |
| **C6. What is the minimum periodontal probing requirement before orthodontic treatment in patients with periodontal disease?** | A.No pockets ≥3mm with BOP (Bleeding on Probing) | **111 (25.58%)** |
|  | ****B. No pockets ≥5mm with BOP**** | ****296 (68.2%)**** |
|  | C. No pockets ≥6mm with BOP | 18 (4.15%) |
|  | D. No pockets ≥7mm with BOP | 9 (2.07%) |
| **C7. Which of the following is NOT recommended during orthodontic treatment in patients with periodontal disease?** | ****A. Patients with severe periodontitis should prioritize using clear aligners**** | ****211 (48.62%)**** |
|  | B. Wire ligation is preferred over elastic ties to reduce plaque accumulation | 58 (13.36%) |
|  | C. Use light and continuous orthodontic forces | 11 (2.53%) |
|  | D. Delay bonding brackets on teeth that will not be adjusted immediately | 154 (35.48%) |
| **C8. Which of the following statements about the frequency of supragingival scaling is correct?** | A. Periodontally healthy patients: every 2 years | 54 (12.44%) |
|  | ****B. Patients with moderate to severe periodontitis: every 3 months**** | ****353 (81.34%)**** |
|  | C. Children undergoing orthodontic treatment: every month | 17 (3.92%) |
|  | D. Patients with peri-implant mucositis: every month | 10 (2.3%) |
| **C9. Which of the following is NOT a periodontal maintenance tool for orthodontic patients?** | A. Water flosser | 1 (0.23%) |
|  | B. Dental floss | 13 (3%) |
|  | C. Interdental brush | 3 (0.69%) |
|  | ****D. Orthodontic wax**** | ****417 (96.08%)**** |
| **C10. What is the first-line treatment for gingival hyperplasia in orthodontic patients?** | A. Immediately stop orthodontic treatment | 7 (1.61%) |
|  | ****B. Enhanced oral hygiene instruction + supragingival scaling**** | ****422 (97.24%)**** |
|  | C. Topical antibiotic ointment application | 1 (0.23%) |
|  | D. Gingivectomy | 4 (0.92%) |
| **C11. Which of the following is NOT part of the routine examination for temporomandibular joint disorders(TMD)?** | A. Mouth opening range and pattern examination | 3 (0.69%) |
|  | B. Psychological status assessment | 132 (30.41%) |
|  | C. Masticatory muscle palpation | 12 (2.76%) |
|  | **D. Lateral cephalometric radiograph** | **287 (66.13%)** |
| **C12. Anterior joint space widening, posterior joint space narrowing, with intact condylar cortex. Which CBCT image corresponds to this description?** | A. | 44 (10.14%) |
|  | ****B.**** | ****363 (83.64%)**** |
|  | C. | 16 (3.69%) |
|  | D. | 11 (2.53%) |
| **C13. Which statement most accurately describes the relationship between orthodontic treatment and TMD?** | A. Orthodontic treatment can prevent most TMD occurrences | 66 (15.21%) |
|  | ****B. There is no clear causal relationship between orthodontic treatment and TMD occurrence**** | ****321 (73.96%)**** |
|  | C. Extraction orthodontics increases TMD risk | 43 (9.91%) |
|  | D. Class II malocclusion inevitably leads to TMD | 4 (0.92%) |
| **C14. Which of the following is the primary treatment principle for TMD?** | A. Surgery as first-line treatment | 1 (0.23%) |
|  | ****B. Conservative treatment as first-line therapy**** | ****399 (91.94%)**** |
|  | C. Occlusal adjustment as first-line treatment | 14 (3.23%) |
|  | D. Orthodontic treatment as first-line therapy | 20 (4.61%) |
| **C15.Which of the following is INCORRECT regarding daily health maintenance for patients with TMD ?** | A. Avoid wide mouth opening | 1 (0.23%) |
|  | B. Heat application to the joint area can relieve muscle pain | 6 (1.38%) |
|  | ****C. Keep teeth tightly clenched to stabilize the joint**** | ****421 (97%)**** |
|  | D. Improve sleep quality and maintain a pleasant mood | 6 (1.38%) |
| **C16. Which of the following myofunctional training exercises is INCORRECTLY matched?** | A. Tongue tip elevation exercises — Lingual muscle function | 7 (1.61%) |
|  | B. Cheek puffing exercises — Buccal muscle function | 10 (2.3%) |
|  | C. Tongue-lip coordination exercises — Swallowing function | 144 (33.18%) |
|  | ****D. Lower lip biting exercises — Orbicularis oris muscle function**** | ****273 (62.9%)**** |
| **C17. In cephalometric analysis, high OSA risk is indicated when posterior airway space (PAS) is less than:** | ****A.11mm**** | ****111 (25.58%)**** |
|  | B.6-10mm | 290 (66.82%) |
|  | C. 5mm | 25 (5.76%) |
|  | D. 3mm | 8 (1.84%) |
| **C18.Which appliance-habit pairing is INCORRECT?** | A. Tongue thrusting habit — Tongue crib | 4 (0.92%) |
|  | B. Mouth breathing — Vestibular screen | 19 (4.38%) |
|  | C. Digit sucking habit — Palatal crib | 31 (7.14%) |
|  | ****D. Unilateral chewing — Lip bumper**** | ****380 (87.56%)**** |
| **C19.A 10-year-old child presents with nocturnal mouth breathing and snoring for 6 months. Physical examination reveals Grade III adenoid hypertrophy. What is the most appropriate initial management?** | **A. Rapid maxillary expansion** | **10 (2.3%)** |
|  | **B. Ear, Nose, and Throat（ENT）referral for evaluation** | **418 (96.31%)** |
|  | C. Vestibular screen appliance therapy | 2 (0.46%) |
|  | D. Orofacial myofunctional therapy | 4 (0.92%) |

Note: In this presentation, correct answers are indicated in bold type.
